# Predicting Treatment Outcomes in Glioblastoma: A Risk Score Model for TMZ Resistance and Immune Checkpoint Inhibition

**DOI:** 10.3390/biology14050572

**Published:** 2025-05-20

**Authors:** Nazareno Gonzalez, Melanie Perez Küper, Matias Garcia Fallit, Alejandro J. Nicola Candia, Jorge A. Peña Agudelo, Maicol Suarez Velandia, Ana Clara Romero, Guillermo Agustin Videla-Richardson, Marianela Candolfi

**Affiliations:** 1 Instituto de Investigaciones Biomédicas (INBIOMED, CONICET-UBA), Facultad de Medicina, Universidad de Buenos Aires, Paraguay 2155, 10th floor, Buenos Aires C1121ABG, Argentina; gonzalez.nazareno1@gmail.com (N.G.); melaniepk@hotmail.com (M.P.K.); mati.garciafallit@gmail.com (M.G.F.); alenicola90@gmail.com (A.J.N.C.); jarmando2192@gmail.com (J.A.P.A.); mmauriciosuarez@unicolmayor.edu.co (M.S.V.); aromero@fmed.uba.ar (A.C.R.); 2 Departamento de Fisiología, Biología Molecular y Celular, Facultad de Ciencias Exactas y Naturales, Universidad de Buenos Aires, Buenos Aires C1428AQK, Argentina; 3 Fundación Para la Lucha Contra las Enfermedades Neurológicas de la Infancia (FLENI), Buenos Aires C1121A6B, Argentina; gvidela@fleni.org.ar

**Keywords:** glioblastoma, immune microenvironment, differentially expressed genes, risk score model, temozolomide resistance

## Abstract

Glioblastoma (GBM) presents significant therapeutic challenges due to its invasive nature and resistance to standard chemotherapy, i.e., temozolomide (TMZ). This study aimed to identify gene signatures that predict poor TMZ response and high PD−L1/PD−1 tumor expression, and explore potential sensitivity to alternative drugs. We analyzed The Cancer Genome Atlas (TCGA) biopsy data to identify differentially expressed genes (DEGs) linked to these characteristics. Among 33 upregulated DEGs, 5 were significantly correlated with overall survival. A risk score model was built using these 5 DEGs, classifying patients into low-, medium-, and high-risk groups. We assessed immune cell infiltration, immunosuppressive mediators, and epithelial–mesenchymal transition (EMT) markers in each group using correlation analysis, Gene Set Enrichment Analysis (GSEA), and machine learning. The model demonstrated strong predictive power, with high-risk patients exhibiting poorer survival and increased immune infiltration. GSEA revealed upregulation of immune and EMT-related pathways in high-risk patients. Our analyses suggest that high-risk patients may exhibit limited response to PD−1 inhibitors, but could show sensitivity to etoposide and paclitaxel. This risk score model provides a valuable tool for guiding therapeutic decisions and identifying alternative chemotherapy options to enable the development of personalized and cost-effective treatments for GBM patients.

## Simple Summary

In this study, we focused on understanding how the immune microenvironment contributes to chemoresistance in glioblastoma (GBM), particularly in patients with poor response to standard chemotherapy, i.e., temozolomide. Using data from these patients, we identified specific genes associated with treatment resistance and developed a risk score model to classify GBM patients based on their prognosis. Stratifying patients using this model revealed a strong correlation between poor survival outcomes and more aggressive tumor characteristics, emphasizing its relevance in predicting aggressive tumor behavior. Notably, patients in the high-risk group exhibited increased levels of immune cells, a feature that may not necessarily be advantageous. While elevated immune cell infiltration is often considered a sign of immune activation, this increased immune activity may lead to a chronic inflammatory state, potentially resulting in lymphocyte exhaustion and impaired immune function. Our strategy aims to leverage the genetic and immune landscape of each individual tumor to guide treatment decisions, selecting drugs with predicted high efficacy while avoiding exposure to highly toxic and potentially ineffective therapies. By refining treatment selection, our research aims to improve outcomes for GBM patients and to address a critical unmet need in cancer therapy.

## 1. Introduction

Glioblastoma (GBM) is the most common and aggressive primary brain tumor in adults, accounting for 14.2% of all primary central nervous system (CNS) tumors and 50% of all malignant primary CNS tumors, with an annual age-adjusted incidence rate of 3.26 per 100,000 population [1]. It is characterized by its rapid growth, diffuse infiltration, and a high recurrence rate, leading to a median survival of approximately 15 months from diagnosis [2]. Despite advancements in surgical techniques, radiotherapy, and chemotherapy, GBM remains particularly difficult to treat. Temozolomide (TMZ), an oral alkylating agent, has been a keystone of GBM treatment since its approval in 2005 [3]. However, nearly all patients eventually develop resistance to TMZ and succumb to tumor progression.

Recent research highlights the crucial role of the tumor immune microenvironment (TME) in GBM progression and treatment response [4,5]. GBM TME exhibits a distinct immune profile, including a high density of immune cells and elevated expression of immune checkpoint molecules such as programmed cell death protein-1 (PD−1) and its ligand PD−L1 [6,7]. Although immune checkpoint inhibitors (ICIs) have revolutionized cancer treatment [8], their efficacy in GBM remains limited [9]. One major factor contributing to this limitation is the complex immune-suppressive microenvironment of GBM, which hampers the efficacy of immunotherapy [10]. This gap underscores the need for reliable biomarkers to predict patient responses to ICIs and other therapies, as well as for targeted strategies to overcome resistance mechanisms.

Emerging evidence has also suggested potential crosstalk between DNA damage response pathways, immunosuppressive signaling, and epithelial–mesenchymal transition (EMT) in GBM. These interactions highlight the potential overlap between mechanisms of resistance to TMZ and ICIs. With the advent of high-throughput genomic technologies, numerous biomarkers associated with GBM diagnosis, prognosis, and therapeutic decision-making have been identified. The generation of risk score signatures has become a widely used and powerful approach for predicting patient outcomes and guiding treatment strategies. Models developed for breast cancer [11,12], lung cancer [13,14], and melanoma [15,16] have demonstrated the utility of integrating multi-dimensional data to stratify patients based on their risk profiles. Our study builds on this foundation by developing a novel risk score model specifically tailored for GBM. Unlike existing models, our risk score not only incorporates general prognostic factors but also addresses specific critical conditions associated with poor prognosis in GBM, including poor response to TMZ and overexpression of PD−1 and PD−L1 [6,17,18]. We believe that understanding dual resistance, involving both TMZ and immune checkpoint inhibitors, may be crucial, particularly given the increasing use of ICIs in GBM trials. By including these factors, our model aims to provide a more accurate and practical risk assessment, helping to guide more effective and personalized treatment strategies for GBM patients.

To develop this model, we classified GBM patients in the TCGA GBM dataset based on three criteria: high TMZ resistance, high expression levels of PD−L1, and high expression levels of PD−1. Thus, we developed our model by leveraging genomic and transcriptomic data to identify differentially expressed genes (DEGs) with prognostic and therapeutic relevance. Shared DEGs represent potential key regulators of both TMZ resistance and immune evasion, forming the basis for our dual resistance model. This distinction is crucial, as our study does not categorize patients directly into a dual resistance group but instead highlights a set of genes that may underlie resistance to both therapies. Our analysis identified five key DEGs—*COL6A3*, *CD163*, *ABCC3*, *COL3A1*, and *THBS1*—that were significantly associated with patient outcomes. By assigning weights to these DEGs, we created a risk score model that stratifies patients into distinct risk groups, reflecting their overall prognosis and potential response to therapy. The model effectively stratified patients into high-, medium-, and low-risk groups; i.e., patients in the high-risk group showed significantly worse overall survival, while those in the medium- and low-risk groups exhibited progressively better outcomes. Further analyses revealed a significant correlation between the risk score and key components of the immune TME, such as immune cell infiltration, as well as oncogenic features involved in tumor progression. Additionally, this model demonstrated strong predictive power for response to immune checkpoint inhibitors and potential alternative therapeutic options, offering a promising tool to guide treatment decisions and improve outcomes for patients with poor prognoses.

## 2. Materials and Methods

### 2.1. Data Collection and Processing

Clinical, genomic, and transcriptomic data for glioma patients were obtained from The Cancer Genome Atlas (TCGA) GBM cohort. We reclassified TCGA-GBM cohort patients based on the latest World Health Organization (WHO) classification of central nervous system (CNS) tumors [19]. This updated classification stratifies glioma patients based on the mutational status of the isocitrate dehydrogenase (IDH) enzyme into two categories: IDH-mutant (mIDH) and IDH wild-type (wtIDH). Since glioblastoma (GBM) is now specifically defined as IDH wild-type, we retained only patients with this genetic profile for our analysis (*n* = 208 patients, *n* male patients = 128, *n* female patients = 80). Additionally, we included only patients aged 30 years and older to align with the typical demographic affected by GBM. Data from The Chinese Glioma Genome Atlas (CGGA) were used as a validation cohort (*n* = 96 GBM patients; *n* male patients = 62; *n* female patients = 34).

In addition, two independent Gene Expression Omnibus (GEO) datasets were used for further validation: GSE53733, which includes transcriptomic profiling of long-term GBM survivors [20], and GSE43378, which includes gene-expression-based prognostic data from GBM and mIDH glioma patients [21]. The results of the validation analyses using these cohorts are presented in Supplementary Figure S5 and described in the Supplementary Materials.

Predictive half-maximal inhibitory concentration (IC50) values for 272 drugs were retrieved from the CancerRxTissue platform to analyze drug response for the TCGA GBM patients [22]. Gene expression data for immune checkpoint markers, including CD274 (encoding PD−L1) and PDCD1 (encoding PD−1), as well as data on canonical targetable signaling pathways [23], were obtained through the UCSC Xena Browser [24].

### 2.2. Differential Expression Analysis

GBM patients were classified into distinct groups based on three criteria: response to temozolomide (TMZ), PD−L1 expression, and PD−1 expression. For TMZ response, patients were stratified into ‘high resistance’ and ‘low resistance’ groups based on data obtained from the CancerRxTissue database [22]. High-PD−L1- and high-PD−1-expression groups were defined using median expression levels as cut-offs. Gene expression profiles were compared between groups to identify differentially expressed genes (DEGs). DEGs were identified using the Xena Differential Gene Expression Analysis Pipeline and extracted using a log fold change (logFC) threshold of 1.5 and an adjusted *p*-value threshold of 0.05. Upregulated genes were included to ensure comprehensive analysis (Supplementary Material—Differential Expression Analysis).

### 2.3. Identification of Prognostic Genes

The prognostic significance of the identified DEGs was assessed using Cox proportional hazards regression analysis. Univariate Cox regression was performed on each gene to evaluate its association with overall survival in GBM patients. Hazard ratios (HRs), 95% confidence intervals (CIs), and *p*-values were computed for each gene. Genes with a *p*-value < 0.05 were considered statistically significant.

Multivariate Cox regression analysis was applied to the DEGs using a stepwise selection method to identify a risk score model that independently predicted prognosis. The final multivariate model included five genes: Collagen Type VI Alpha 3 Chain (*COL6A3*), Cluster of Differentiation 163 (*CD163*), ATP-Binding Cassette Subfamily C Member 3 (*ABCC3*), Collagen Type III Alpha 1 Chain (*COL3A1*), and Thrombospondin-1 (*THBS1*). Hazard ratios were calculated to assess the impact of each gene on survival.

### 2.4. Establishment of the Risk Score Model for Prognosis and Treatment Response Prediction

Gene expression data were normalized using log2 transformation and z-score normalization to ensure consistency across datasets. Based on the DEGs identified through multivariate Cox proportional hazards regression analysis, we developed a risk score model using the following formula:

Risk Score = Σ (β_i_ × E_i_), for i = 1 to *n*

In this formula, “*n*” represents the total number of key genes, “β_i_” is the regression coefficient for gene i, and “E_i_” represents the expression level of gene i. The “βi” values used in the validation CGGA datasets were the same as those derived from the TCGA cohort.

To evaluate the prognostic and predictive value of the risk score, GBM patients were stratified into high-risk, medium-risk, and low-risk groups based on the risk score cut-off determined by quartile analysis. Univariate and multivariate Cox regression analyses were performed on clinicopathological parameters and the risk score to confirm the clinical significance of the model.

### 2.5. Kaplan–Meier Survival Analysis

Kaplan–Meier survival analyses were conducted to compare overall survival (OS) between the high-, medium-, and low-risk groups. Survival curves were plotted to illustrate the prognostic power of the gene signature.

### 2.6. Predictive Effect for Alternative Drugs According to the Risk Score

Data on the predicted IC50 values of 272 drugs for TCGA glioma patients were obtained from the CancerRxTissue database [22]. TCGA GBM patients were stratified into three risk score groups: “low”, “medium”, and “high”. The predicted IC50 values for temozolomide (TMZ) and several other chemotherapeutic drugs were then evaluated across these groups. To validate the predictive results, in vitro testing was performed using the MTT assay to assess cell viability in TMZ-resistant (TMZ-r) and control-sensitive GBM cells at fixed concentrations of the drugs.

### 2.7. Clinical Response to Anti-PD1 Therapies: Model Development

Data on clinical response to anti-PD−1 therapies and associated gene expression profiles were obtained from Zhao et al. (nivolumab response in GBM) [25] and Cloughesy et al. (pembrolizumab response in GBM) [26]. A machine learning model was developed to predict patient responses to anti-PD−1 inhibitors (responders vs. non-responders) using the expression levels of five differentially expressed genes (DEGs) identified during risk score construction: *COL6A3*, *CD163*, *ABCC3*, *COL3A1*, and *THBS1*.

The model was trained using LightGBM, a gradient boosting framework recognized for its efficiency and accuracy in handling classification problems involving large datasets. Hyperparameter optimization was performed using RandomizedSearchCV, exploring parameters such as num_leaves, n_estimators, learning_rate, and subsample. The optimal hyperparameters were subsequently employed to train the final LightGBM model.

The performance of the model was evaluated using standard classification metrics, including accuracy, precision, recall, F1 score, and area under the receiver operating characteristic curve (ROC-AUC). The ROC-AUC score was specifically calculated to assess the model’s ability to distinguish responders from non-responders. Additionally, Kaplan–Meier survival analysis was conducted to evaluate the association between predicted response groups and overall survival in the independent validation dataset derived from TCGA GBM data.

### 2.8. Drugs

Temozolomide (TMZ) was obtained from Sigma (St. Louis, MO, USA). Paclitaxel was purchased RhenochemAG (Basel, Switzerland). Cisplatin and etoposide were obtained from Microsules Argentina (Buenos Aires, Argentina), and nivolumab was acquired from Laboratorio Elea Phoenix (Buenos Aires, Argentina). Dimethyl sulfoxide (DMSO) was obtained from Ciccarelli (Córdoba, Argentina).

### 2.9. Cell Culture Reagents

Dulbecco’s Modified Eagle Medium (DMEM; Cat# 12100046), penicillin–streptomycin (Cat# 15140122), and trypsin–EDTA (0.025%, Cat# 25200114) were obtained from Gibco (Invitrogen, Carlsbad, CA, USA). Fetal bovine serum (FBS) was acquired from Natocor (Cordoba, Argentina).

### 2.10. Cell Culture

Human GBM commercial cell lines (U-251 and U-87) were kindly donated by Dr Maria G Castro (University of Michigan School of Medicine, Ann Arbor, MI, USA) and maintained routinely in DMEM supplemented with 5% FBS and 1% penicillin–streptomycin (PS), pH 7.4, under 5% CO2 atmosphere and 37 °C. Once the cells reached 80% confluence, they were dissociated with 0.05% trypsin–EDTA and subcultured in 100 mm plastic Petri dishes every three days.

### 2.11. Generation of TMZ-Resistant Cell Lines

To generate TMZ-resistant U-251 and U-87 cell lines, cells were exposed to increasing concentrations of TMZ, ranging from 15 µM to 100 µM. Each treatment cycle lasted 72 h, followed by a 24 h recovery period. After completing the concentration gradient, the cell lines were maintained by weekly 72 h treatments with 100 µM TMZ to preserve the resistant phenotype.

### 2.12. MTT Cell Viability Assay

To evaluate the therapeutic effect of chemotherapeutic drugs (TMZ, etoposide, paclitaxel, and cisplatin) and immune checkpoint inhibitors (ICIs; nivolumab) based on our risk score model in control and TMZ-resistant cell lines, we performed a 3-(4,5-dimethylthiazol-2-yl)-2,5-diphenyltetrazolium bromide (MTT) cell viability assay.

For the assay, each cell line was seeded at a density of 5000 cells per well in 96-well plates. After 24 h, cells were washed and treated with 100 µL of the respective agents at fixed concentrations (diluted in the previously described culture medium). The treatments included temozolomide (TMZ; 15 µM), etoposide (0.5 µM), paclitaxel (10 nM), cisplatin (5 µM), nivolumab (50 µg/mL), avelumab (50 µg/mL), and vehicle control. Following 72 h of incubation, the treatment medium was removed, and the wells were washed. Subsequently, 110 µL of MTT solution (450 µg/mL; Molecular Probes, Invitrogen, Thermo Fisher Scientific, Waltham, MA, USA) prepared in Krebs–Henseleit buffer was added to each well. The plates were incubated for 4 h at 37 °C. After the incubation period, the MTT–Krebs solution was carefully discarded, and 100 µL of a solution containing 0.04 M HCl in isopropanol was added to each well to dissolve the formazan precipitate. The absorbance was then measured at 595 nm using a spectrophotometer.

The concentration of 15 µM was selected based on previous studies demonstrating its efficacy in GBM cell lines, including those with TMZ resistance [27]. Additionally, we included a concentration–response curve for each chemotherapeutic drug used in Supplementary Figure S1 and described in the Supplementary Materials to further support our concentration selection. The concentration–response curve of temozolomide (TMZ) in U-251 control and TMZ-resistant cell lines is presented in Supplementary Figure S2 and described in the Supplementary Materials.

### 2.13. Flow Cytometry

U-251 control cells and U-251 TMZ-resistant cells were harvested and prepared for flow cytometry analysis. Cells were washed with phosphate-buffered saline (PBS) and detached using 0.025% trypsin–EDTA. The samples were centrifuged at 1500 rpm for 5 min, and the supernatant was discarded. After an additional wash with PBS containing 1% FBS, cells were incubated with anti-PD−1 antibody (Cat# 3299033, BioLegend, United States) and anti-PD−L1 antibody (Cat#393610, BioLegend, USA) at a 1:100 dilution for 30 min in the dark. After incubation, cells were washed with PBS and centrifuged at 1500 rpm for 5 min. The cell pellets were resuspended in 200 µL of PBS and immediately analyzed using a BD FACS Aria II flow cytometer (BD Biosciences, USA).

For each sample, 20,000 events were acquired using the FITC and APC fluorochrome channel. Data analysis was performed using FlowJo™ v10 software (BD Biosciences).

Flow cytometry controls (negative controls, gating strategy, and compensation details) are provided in Supplementary Figure S3 and described in the Supplementary Materials.

### 2.14. Statistical Analyses

Statistical analyses were performed using GraphPad Prism version 8 software (GraphPad Software, version 8). Data normality was assessed using the Kolmogorov–Smirnov test before parametric statistical tests were conducted. Continuous variables were compared using a *t*-test or one-way analysis of variance (ANOVA), as appropriate. Correlations between continuous variables were evaluated using Spearman correlation analysis. Kaplan–Meier curves were analyzed using the log-rank test. Differences were considered significant when the *p*-value < 0.05.

## 3. Results

### 3.1. Identification of Differentially Expressed Genes (DEGs)

**Figure 1:**
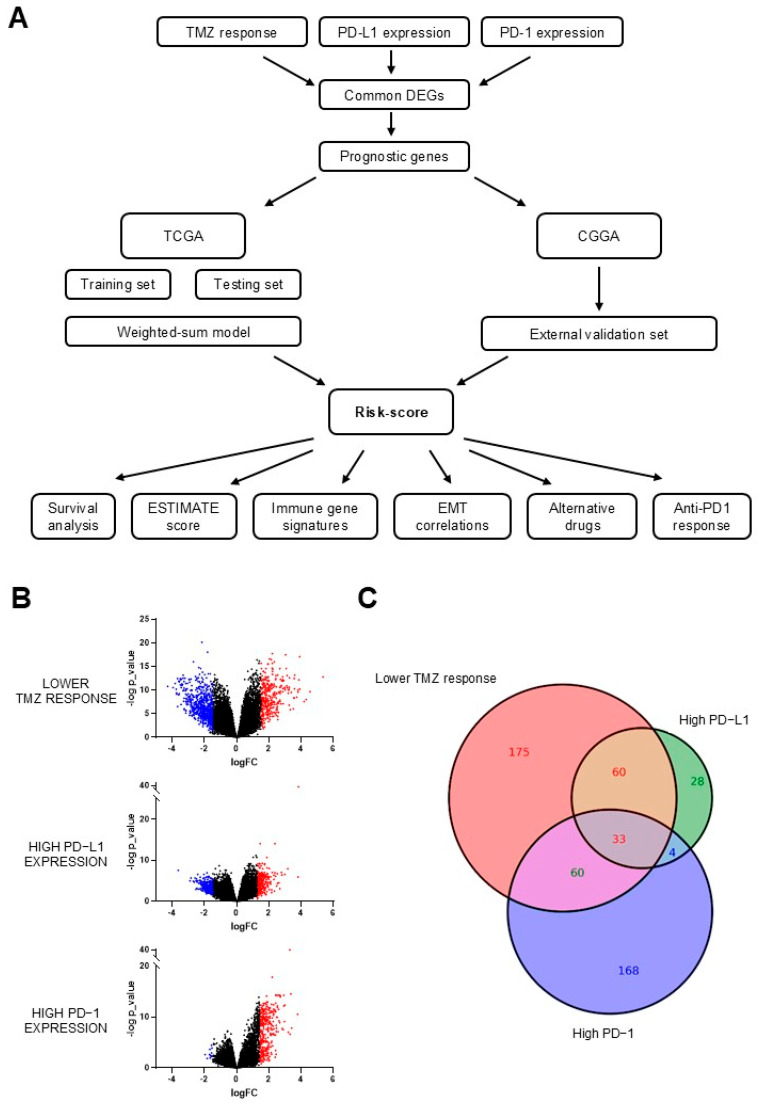
Identification of differentially expressed genes (DEGs) in GBM biopsies with high TMZ resistance and high expression levels of PD−L1 and PD−1. (**A**) The flow chart for constructing and verifying the 5-gene risk score signature. (**B**) Volcano plots showing the differentially expressed genes (DEGs) identified under three specific conditions: lower temozolomide (TMZ) response, high PD−L1 expression, and high PD−1 expression. Genes with a *p*-value below 0.05 and log fold change above 1.5 and below −1.5 are marked with highlighted red and blue dots, respectively. (**C**) Venn diagram displaying the overlap of DEGs across the three conditions, identifying 33 common DEGs that were upregulated in biopsies with low TMZ response and high PD−L1 and PD−1 expression.

Using the TCGA dataset, we classified GBM patients based on three criteria: high TMZ resistance, high expression levels of PD−L1, and high expression levels of PD−1 (Figure 1A, Table S1). We identified DEGs for each classification (Tables S2–S4), and volcano plots were generated to visualize differential gene expression in each group (Figure 1B). A Venn diagram analysis was conducted to identify upregulated DEGs that were overexpressed in all three groups, revealing 33 shared DEGs (Figure 1C).

To assess the prognostic value of these DEGs in GBM, we conducted a univariate Cox regression analysis for each gene, with overall survival as the endpoint. As shown in Table 1, 14 upregulated genes exhibited a significant association with survival (*p* < 0.05), with several genes inversely correlated with patient outcomes.

A subsequent multivariate Cox regression analysis of these 33 DEGs identified a 5-gene signature significantly associated with overall survival: Collagen Type VI Alpha 3 Chain (*COL6A3*), Cluster of Differentiation 163 (*CD163*), ATP-Binding Cassette Subfamily C Member 3 (*ABCC3*), Collagen Type III Alpha 1 Chain (*COL3A1*), and Thrombospondin-1 (THBS1). Among these, *COL6A3*, *ABCC3*, and *THBS1* were associated with poorer survival (hazard ratio (HR) > 1), while *CD163* and *COL3A1* were associated with improved survival (HR < 1). These five genes that encompass the signature represent important markers for overall survival in GBM patients, indicating varying prognostic impacts of these genes.

### 3.2. Construction of the Risk Score Model

**Figure 2:**
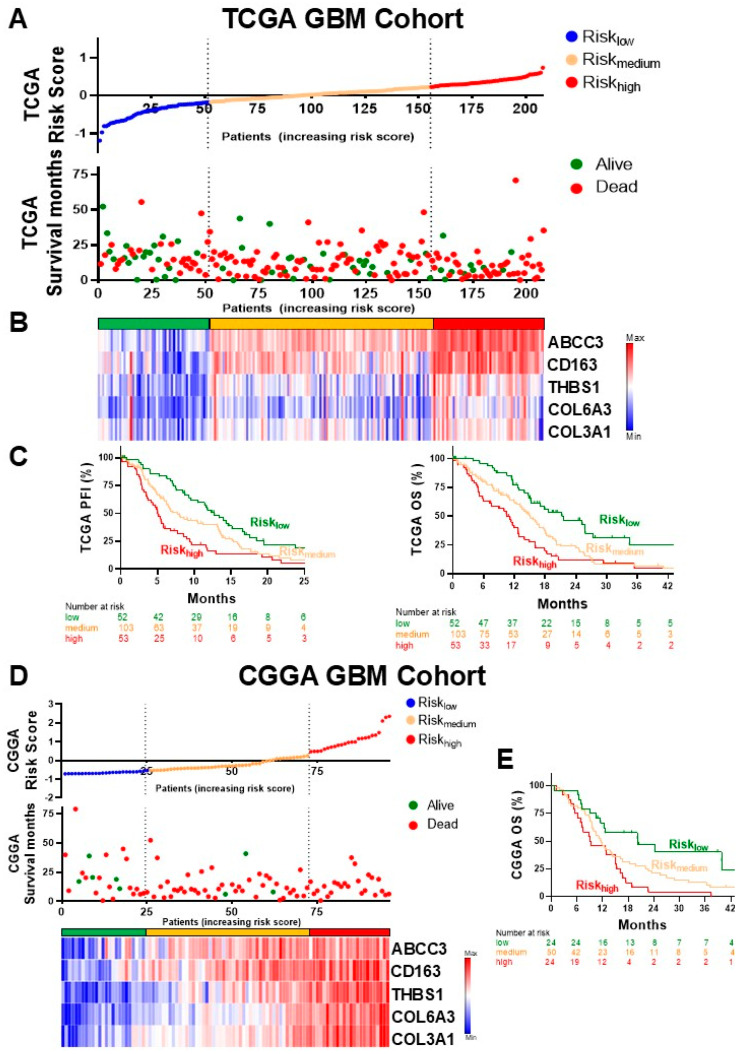
Risk score analysis and survival outcomes in GBM patients. (**A**) Distribution of risk score, survival time, and survival status of TCGA GBM patients. (**B**) Heatmap displaying the expression patterns of the 5 selected genes across the different risk score groups. The color gradient reflects the level of gene expression, with a clear distinction observed across low-, medium-, and high-risk categories. (**C**) Kaplan–Meier survival curves for TCGA GBM patients, stratified by risk score groups, showing the progression-free interval (PFI) and overall survival (OS) (*p* < 0.05, log-rank test). (**D**) Validation of the risk score model using the CGGA GBM patient dataset. This panel includes risk score distribution and survival status scatter plots, a heatmap of the 5 gene expression patterns across risk score groups, and (**E**) Kaplan–Meier survival curves for overall survival (OS) in the validation set, confirming the predictive power of the model (*p* < 0.05, log-rank test).

We next developed a risk score model based on the expression levels of the five DEGs from Table 1 using a weighted sum method. Gene expression data from GBM patients were normalized and standardized. A Cox proportional hazards model was fitted to determine the weights for each gene. Patients were stratified into low-, medium-, and high-risk groups based on quartiles of the risk scores, revealing that patients in the predicted high-risk group exhibited significantly shorter survival times compared to those in the groups with predicted low and medium risk, highlighting the strong correlation between elevated risk scores and poor patient outcomes (Figure 2A).

The Cox proportional hazards model was applied to derive gene weights, and cross-validation confirmed the predictive performance of the model. The final risk score model was calculated as a weighted sum of the expression levels of the five DEGs, with the weights derived from the Cox model coefficients, and the corresponding heatmap expression of the five key genes is shown (Figure 2B). Kaplan–Meier survival analysis demonstrated that patients in the high-risk group had significantly poorer overall survival compared to those in the low- and medium-risk groups (Figure 2C).

The risk score model was further validated using an independent dataset from the Chinese Glioma Genome Atlas (CGGA) to assess its robustness and generalizability (Figure 2D). This external validation confirmed the initial findings observed in the TCGA dataset. Specifically, patients classified into the high-risk group in the CGGA dataset demonstrated significantly worse overall survival compared to those in the low- and medium-risk groups (Figure 2E).

Finally, univariate and multivariate analyses revealed that the risk score is a significant predictor of progression-free interval (PFI) and overall survival (OS), independent of gender, age, and Karnofsky performance score (Table 2).

### 3.3. Correlation with Oncogenic Features

**Figure 3:**
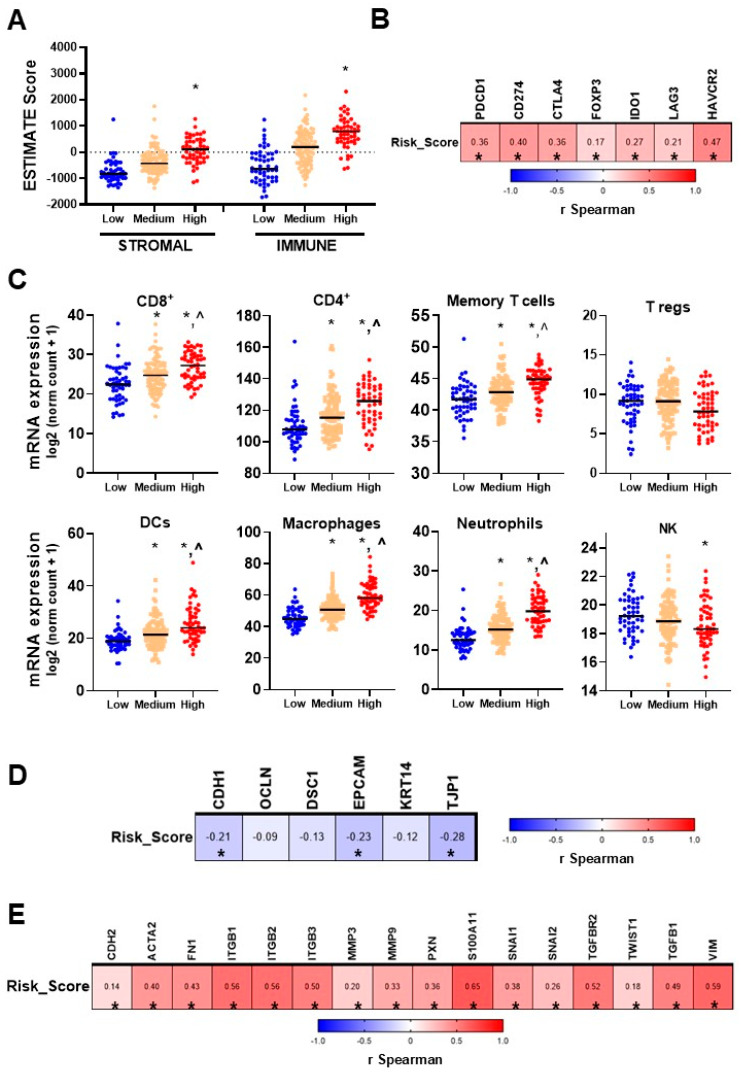
Correlation of risk scores with immune, epithelial, and mesenchymal markers in GBM patients. (**A**) Dot plot showing the relationship between ESTIMATE scores and risk scores, categorized into low, medium, and high groups. (**B**) Spearman correlation analysis between risk scores and immune marker expression. *PDCD1* (PD−1), *CD274* (PD−L1), *CTLA4* (Cytotoxic T-Lymphocyte Antigen 4), *FOXP3* (Forkhead Box P3), *IDO1* (Indoleamine 2,3-dioxygenase), *LAG3* (Lymphocyte-Activation Gene 3), *HAVCR2* (Hepatitis A Virus Cellular Receptor 2). *, *p* < 0.05. (**C**) Dot plot displaying the infiltration gene signatures across different risk score groups (low, medium, high). *, *p* < 0.05 vs. low risk score group; ^, *p* < 0.05 vs. all groups, ANOVA. Spearman correlation analysis between risk scores and (**D**) epithelial marker expression: *CDH1* (E-cadherin), *OCLN* (Occludin), *DSC1* (Desmocollin 1), *EPCAM* (Epithelial Cell Adhesion Molecule), *KRT14* (Keratin 14), *TJP1* (Tight Junction Protein 1). *, *p* < 0.05. (**E**) Mesenchymal marker expression: *CDH2* (N-cadherin), *ACTA2* (Alpha-Smooth Muscle Actin), *FN1* (Fibronectin 1), *ITGB1* (Integrin beta-1), *ITGB2* (Integrin beta-2), *ITGB3* (Integrin beta-3), *MMP3* (Matrix Metalloproteinase 3), *MMP9* (Matrix Metalloproteinase 9), *PXN* (Paxillin), *S100A11* (S100 Calcium Binding Protein A11), *SNAI1* (Snail Family Transcriptional Repressor 1), *SNAI2* (Snail Family Transcriptional Repressor 2), *TGFBR2* (Transforming Growth Factor Beta Receptor 2), *TWIST1* (Twist Family BHLH Transcription Factor 1), *TGFB1* (Transforming Growth Factor Beta 1), *VIM* (Vimentin). *, *p* < 0.05.

The risk score was analyzed in relation to various oncogenic features using data from the TCGA GBM cohort, revealing significant associations with key immune markers and immune infiltrate gene signatures. Analyzing ESTIMATE scores, a computational method to infer the levels of stromal and immune cell components in tumor samples based on gene expression data, demonstrated a positive correlation between the risk score and ESTIMATE scores, indicating that patients in the higher risk group would exhibit increased stromal and immune cell components in the TME (Figure 3A). Furthermore, the risk score showed a positive and statistically significant correlation with several immune markers commonly associated with immunosuppression. Specifically, the risk score was significantly correlated with the expression levels of PD−L1, PD−1, CTLA4, IDO1, LAG3, and TIM3 in the biopsies of GBM patients (Figure 3B).

Analysis of immune infiltrate gene signatures [28,29] revealed distinct cell population dynamics between risk groups. In high-risk patients, we found elevated levels of gene signatures for CD8+ T cells, CD4+ T cells, dendritic cells, and macrophages, suggesting augmented immune cell infiltration in the tumor, a characteristic of a more inflammatory tumor microenvironment and potential immunosuppression. Conversely, lower levels of Natural Killer (NK) cells were observed in the high-risk group (Figure 3C).

Additionally, a positive correlation was observed between the risk score and EMT markers, implying that a high risk score is associated with a mesenchymal phenotype. Significant statistical correlation was detected for epithelial markers (*CDH1*, *OCLN*, *EPCAM*, *KRT14*, *TJP1*) (Figure 3D) and mesenchymal markers (*CDH2*, *ACTA2*, *FN1*, *ITGB1*, *ITGB2*, *ITGB3*, *MMP3*, *MMP9*, *PXN*, *S100A11*, *SNAI1*, *SNAI2*, *TGFBR2*, *TWIST1*, *TGFB1*, *TGFB2*, *VIM*) (Figure 3E). These findings underscore the potential of the risk score to predict the characteristics of the tumor microenvironment and the immunological profile of the tumor.

### 3.4. GSEA Analysis

**Figure 4:**
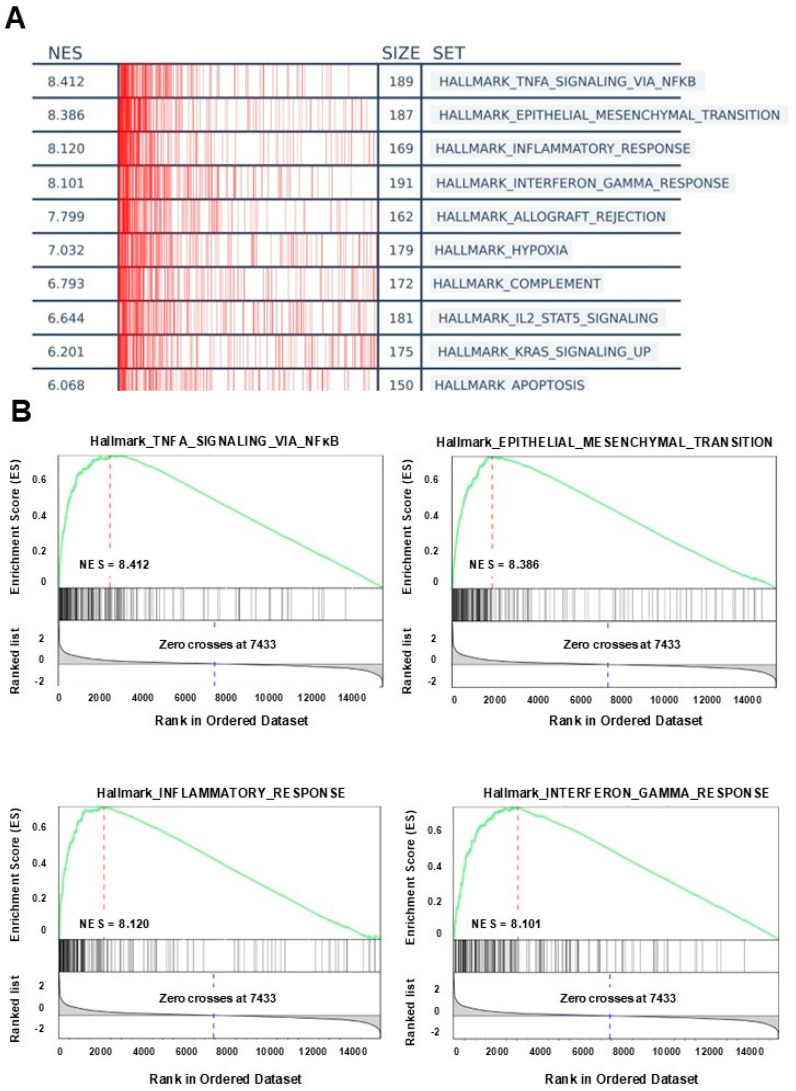
Genome Enrichment Analysis (GSEA) in high risk score group. (**A**) Genome Enrichment Analysis (GSEA) (hallmark) for the high risk score group. The top 10 enriched gene sets are presented, ranked by Normalized Enrichment Score (NES), including TNFα signaling via NFκB, epithelial–mesenchymal transition, interferon-gamma response, and inflammatory response. (**B**) Representative enriched pathways in high-risk GBM through GSEA analysis.

We conducted a Gene Set Enrichment Analysis (GSEA) to identify pathways associated with the five-gene signature in high-risk versus low-risk patients. The analysis revealed significant enrichments in several key biological pathways, reinforcing the robustness of the risk score model. Notably, high risk scores were strongly associated with immune-related pathways, including TNF-α signaling via NFκB, interferon-gamma response, and inflammatory response, as well as EMT hallmarks. These findings underscore the prominent role of immune activation and inflammatory signaling, alongside EMT, in high-risk glioma patients (Figure 4A,B). Detailed results of the differential expression analysis for risk-high versus risk-low patients and the GSEA results are presented in the Supplementary Material (Table S5).

### 3.5. Prediction of Drug Sensitivity According to the Risk Score

To identify potential drug alternatives to TMZ for GBM patients, we explored correlations between the predicted ln(IC50) values of drugs from CancerRxTissue [22] and risk score levels in biopsies from GBM patients. TCGA GBM patients were classified into “low”, “medium”, and “high” risk score groups, and the predicted ln(IC50) values for TMZ and several alternative chemotherapeutic drugs were evaluated.

**Figure 5:**
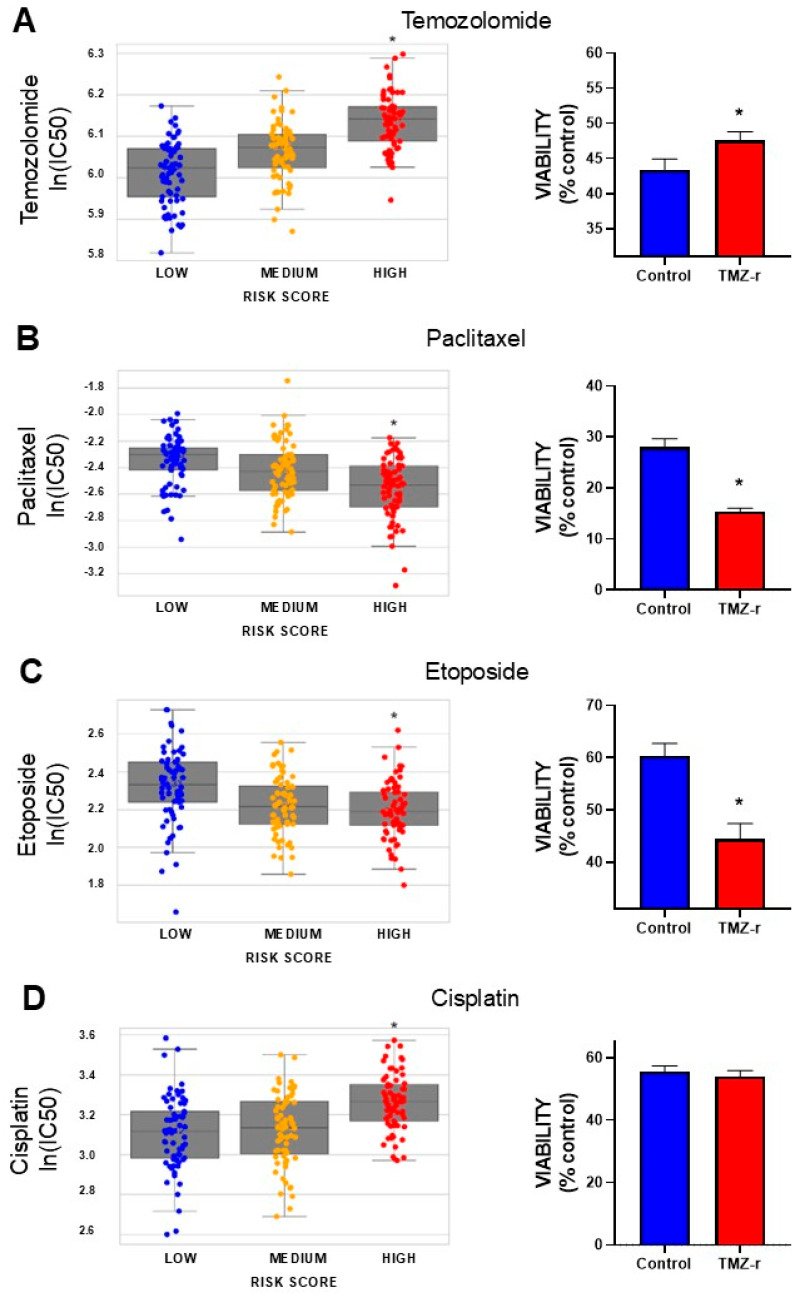
Prediction of drug sensitivity according to the risk score. Left panels show box plots depicting the predicted sensitivity (IC50 values) of GBM patients to chemotherapeutic drugs, namely (**A**) temozolomide (TMZ), and alternative drugs like (**B**) paclitaxel, (**C**) etoposide, and (**D**) cisplatin, according to their risk score. Spearman correlation, * *p* < 0.05. The right panels show the corresponding in vitro testing in TMZ-resistant (TMZ-r) or control U-251 GBM cells. Cells were exposed to TMZ (15 µM), paclitaxel (10 nM), etoposide (0.5 µM), and cisplatin (5 µM). Cell viability was measured 72 h post-treatment using the MTT assay. The viability of each type of cell is shown as the percentage of viable cells compared to untreated cells *, *p* < 0.05, *t*-test.

We observed a positive correlation between predicted TMZ ln(IC50) values and risk scores, confirming that patients with elevated risk scores are less sensitive to TMZ (Figure 5A). We next evaluated the predicted effects of several chemotherapeutic drugs based on risk score levels. We identified a negative correlation between the ln(IC50) values of etoposide and paclitaxel and risk scores in GBM biopsies, indicating that these drugs may be effective in patients who do not respond to TMZ. In contrast, we found a positive correlation between the ln(IC50) values of cisplatin and risk scores, suggesting that this treatment may not benefit high-risk patients (Figure 5A).

To validate these findings, we assessed the response of U-251 TMZ-resistant GBM cells (TMZ-r) to these drugs. Consistent with our predictions, we found that TMZ and cisplatin showed no significant effect in TMZ-r cells. In contrast, etoposide and paclitaxel exhibited significantly stronger antitumoral effects in TMZ-r cells compared to control cells, confirming that these drugs may provide potential alternatives for patients with high-risk profiles and TMZ resistance.

### 3.6. Prediction of Clinical Response to Anti-PD−1 Inhibitors

A machine learning model was developed to predict clinical responses to PD−1 inhibitors based on the expression levels of five significant common DEGs identified from the weighted sum model. The analysis focused on distinguishing responders and non-responders among GBM patients using gene expression data and clinical response from two different clinical trials extracted from ClinicalOmics [30]. These studies included a clinical trial assessing neoadjuvant anti-PD−1 immunotherapy with pembrolizumab (n = 35) [26] and a retrospective series of 66 adult GBM patients treated with PD−1 inhibitors (pembrolizumab or nivolumab) upon recurrence [25]. The model was trained using LightGBM with hyperparameters optimized through Randomized Search CV. The best parameters were identified as ‘subsample’: 0.8, ‘num_leaves’: 63, ‘n_estimators’: 200, ‘learning_rate’: 0.1, ‘colsample_bytree’: 1.0, ‘boosting_type’: ‘dart’. This configuration achieved an accuracy score of 80% on validation data, indicating robust predictive capability. To ensure the selection of the most effective model, a comparative analysis was conducted, evaluating Random Forest and Support Vector Machines (SVMs) alongside LightGBM. All models underwent similar hyperparameter optimization. The results revealed that LightGBM outperformed both Random Forest and SVMs, demonstrating the highest AUC-ROC (0.90), the best balance of precision and recall, and the most robust MCC and Cohen’s Kappa scores. A detailed table of the performance metrics, including accuracy, precision, recall, F1 score, AUC-ROC, MCC, Cohen’s Kappa, and Log Loss, is provided in the Supplementary Materials (Prediction of Clinical Response to Anti-PD−1 Inhibitors).

**Figure 6:**
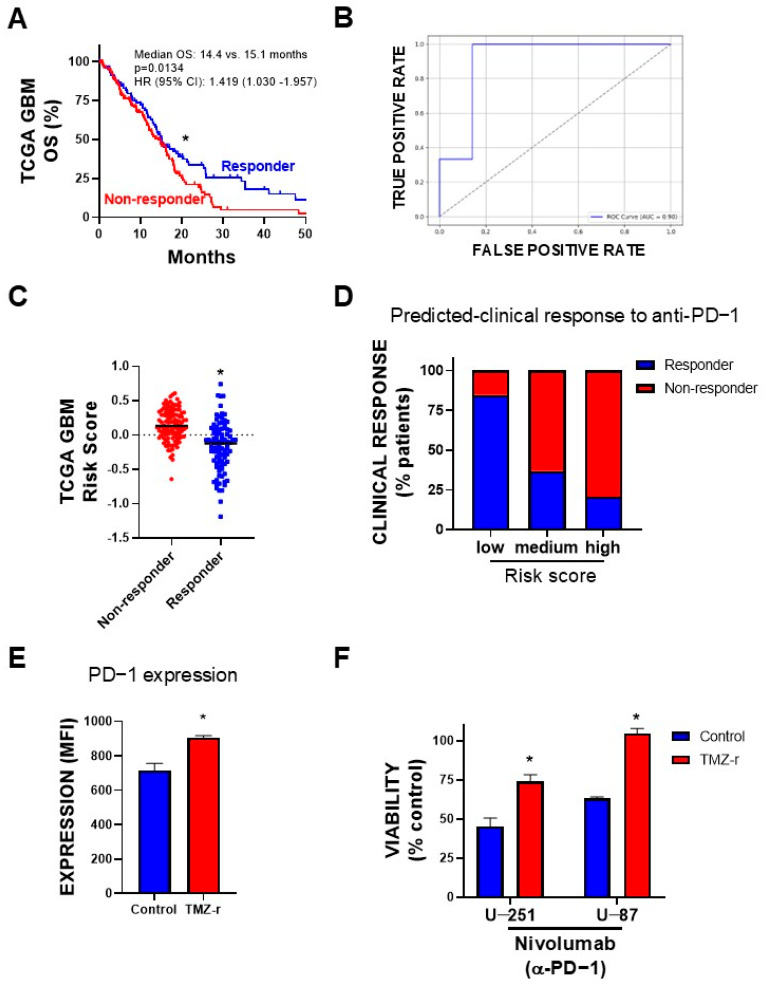
Prediction of clinical response to anti-PD−1 inhibitors. (**A**) Kaplan–Meier survival curves for predicted responders and non-responders to anti-PD−1 inhibitors in TCGA GBM patients, based on the machine learning model developed. The curves show significantly lower overall survival for predicted non-responders compared to responders (*p* < 0.05, log-rank test). (**B**) ROC-AUC curve demonstrating the performance of the machine learning model in discriminating between responders and non-responders (AUC = 0.90). (**C**) Distribution of risk scores between predicted responders and non-responders to PD−1 inhibitors in TCGA GBM patients. *, *p* < 0.05; unpaired *t*-test. (**D**) Distribution of PD−1 inhibition responders and non-responders within each risk score group: low-risk group (84.6% responders, 15.4% non-responders), medium-risk group (36.9% responders, 63.1% non-responders), and high-risk group (20.7% responders, 79.2% non-responders). (**E**) PD−1 expression was evaluated in TMZ-sensitive and TMZ-resistant (TMZ-r) U-251 glioblastoma cell lines using flow cytometry. The expression of PD−1 was significantly increased in the TMZ-r GBM cell line compared to the sensitive counterpart. Data are represented as mean fluorescence intensity (MFI). (**F**) U-251 and U-87 TMZ-r cells were treated with an anti-PD−1 antibody (nivolumab, 50 µg/mL). Cell viability was measured 72 h post-treatment using the MTT assay. Statistical significance was determined using a *t*-test (* *p* < 0.05 vs. corresponding control).

The model was subsequently applied to predict responses in an independent dataset of TCGA GBM patients. Kaplan–Meier analysis of these patients validated the prediction model, revealing that patients with predicted resistance to PD−1 inhibitor therapy had significantly lower overall survival (Figure 6A). The ROC-AUC curve, which illustrates the ability of the model to discriminate between responders and non-responders, showed an area under the curve (AUC) of 0.90 (Figure 6B). Full performance metrics and additional details can be found in the Supplementary Materials (Prediction of Clinical Response to Anti-PD−1 Inhibitors).

Interestingly, patients with high risk scores may also exhibit limited or no response to anti-PD−1 treatment (Figure 6C). The distribution of predicted responders to PD−1 inhibition within each risk group was as follows: low-risk group: 84.6%; medium-risk group: 36.9%; and high-risk group: 20.7% (Figure 6D).

Considering that it has been previously shown that PD−1 can be expressed in tumor cells [31,32,33], we evaluated its expression levels in both control (U-251 CTRL) and TMZ-resistant (U-251 TMZ-r) cells (Figure 6E). Our analysis revealed that U-251 TMZ-r cells exhibited increased levels of PD−1 compared to the control cells.

Given that PD−1 effects may not only be immune-mediated but could also exert tumor-intrinsic effects [17], we evaluated the effect of nivolumab (anti-PD−1) in U-251 and U-87 TMZ-r GBM cells. We found that this monoclonal antibody elicited direct antitumor effects in these GBM cells (Figure 6F). Consistent with our machine learning predictions, we found that nivolumab exhibited significantly reduced efficacy in both TMZ-r cells (Figure 6F), aligning with the predicted non-responder profile of high-risk patients. Interestingly, when we treated these cells with an anti-PD−L1 antibody (avelumab), which may not inhibit receptor activation, no significant effect on cell viability was observed (Supplementary Figure S4), despite elevated PD−L1 expression in the TMZ-r cells. These findings support the specificity of the anti-PD−1 effect and suggest that anti-PD−1 inhibitors may be less effective in patients with high-risk profiles and resistance to TMZ.

## 4. Discussion

Our study introduces a novel risk score model for GBM, using genomic and transcriptomic data to address critical factors associated with poor prognosis, including high resistance to TMZ and an immunosuppressive tumor microenvironment. The genes that compose the risk signature—*COL6A3*, *CD163*, *ABCC3*, *COL3A1*, and *THBS1*—are not only linked to patient outcomes, but also reflect the complex biology of GBM. COL6A3 and COL3A1 are involved in extracellular matrix (ECM) remodeling, which is crucial for tumor progression. These genes encode proteins that are known to influence tumor cell adhesion, migration, and invasion [34,35,36]. The overexpression of these collagens in GBM highlights their role in creating a supportive environment for tumor growth and invasion. CD163 serves as a marker for macrophage polarization, particularly in tumor-associated macrophages (TAMs) [37]. While some studies have shown *CD163* to be upregulated and associated with poor prognosis in GBM [38,39], its role in GBM progression remains controversial, with evidence suggesting it may also be linked to better outcomes under certain conditions. ABCC3, a member of the ATP-binding cassette (ABC) transporter family, is known for its role in drug resistance in many types of tumors [40,41,42,43], including GBM, where it is overexpressed and correlates with tumor progression, worse prognosis [44,45], and a lower response to TMZ [46]. Finally, THBS1, or Thrombospondin-1, is a multifunctional protein that not only acts as an endogenous inhibitor of angiogenesis, but also promotes tumor invasion, metastasis, and immune response in the tumor environment [47]. In GBM, *THBS1* overexpression was found to be associated with poor overall survival [48]. Together, these DEGs control critical aspects of GBM biology, i.e., its ability to remodel its microenvironment, evade immune surveillance, and develop treatment.

To create our risk score signature, we aimed to select genes that show strong and statistically significant associations with overall survival, particularly in the multivariate analysis, as this accounts for potential confounders. Interestingly, the multivariate analysis revealed that the genes included in our risk model exhibit hazard ratios (HRs) that are above 1, i.e., *COL6A3*, *THBS1*, and *ABCC3*, but also genes with HRs below 1, CD163 and COL3A1, which may exert a protective effect or a less direct role in prognosis. In fact, the role of CD163 and COL3A1 remains controversial. The expression of *CD163* is associated with tumor-associated macrophages (TAMs), and thus, it has also been identified as a marker of worse prognosis in GBM [37,38,39]. In addition, while high expression of *COL3A1*, along with SNAP91, has been proposed to confer a survival advantage in GBM patients [49], others have associated high *COL3A1* expression with significantly poorer outcomes in GBM [34]. However, these conclusions were derived solely from univariate analyses, which assessed *CD163* or *COL3A1* as isolated markers of overall survival. In contrast, our findings stem from a multivariate approach that evaluates these genes as part of a gene signature, highlighting the importance of considering their role within a broader biological context. Additionally, these previous studies did not classify patients based on the latest WHO classification of CNS tumors [19], which could further influence the interpretation of the prognostic role of *CD163* and *COL3A1*. Including a mix of genes with HRs above and below 1 provides a balanced and robust view of the biological behavior of the tumor. This approach addresses a common limitation in some gene signatures [50,51], which rely exclusively on genes with HRs greater than 1. Focusing solely on such genes assumes that only those associated with poor outcomes are relevant, potentially overlooking protective or beneficial factors that contribute to a more comprehensive and predictive model. This increases the predictive power of the model and its ability to better stratify patients for personalized treatments. Furthermore, the multivariate analysis shows that our risk score signature is the most critical variable, among gender, age, and Karnofsky performance score, predicting both progression and survival outcomes in the TCGA cohort, making these five genes potentially valuable biomarkers for further investigation.

High-risk patients exhibit an upregulation of immune gene signatures, i.e., CD4+ and CD8+ T cells, dendritic cells (DCs), macrophages, and neutrophils, which aligns with the fact that increased infiltration of macrophages and neutrophils is characteristic of persistent chronic inflammation that drives pro-tumorigenic effects [52]. Interestingly, unlike in most solid tumors, higher levels of tumor-infiltrating lymphocytes (TILs) in GBM are associated with worse outcomes [29,53,54,55]. Additionally, the positive correlation of the risk score with immune checkpoints suggests that many lymphocytes in these tumors may be in an exhausted state. Moreover, significant enrichment was found in immune-related and inflammatory pathways in the tumors of high-risk patients. The observed correlation between the risk score and *SNAI1*/*SNAI2* expression aligns with the enrichment of the TNF-α/NFκB signaling pathway in high-risk patients, a pathway that has been implicated in cancer cell migration and invasion through activation of Snail family transcription factors, as previously demonstrated in other malignancies [56]. Furthermore, the positive NES for the hallmark interferon-gamma response in high-risk patients highlights the association between immune activation and GBM progression. This finding is consistent with recent evidence demonstrating upregulation of the canonical interferon-gamma signaling pathway in GBM and its correlation with worse outcomes [57]. Additionally, WNT/β-catenin signaling has emerged as a key pathway in GBM progression and therapy resistance. Aberrant activation of this pathway has been linked to increased tumor invasiveness and TMZ resistance, in part due to its role in regulating glioma stem-like cell populations [58,59]. Furthermore, STING (stimulator of interferon genes) pathway alterations have been identified as a mechanism affecting TMZ efficacy in GBM. STING-mediated immune activation has been shown to enhance the therapeutic response to TMZ, particularly in tumors harboring PTEN mutations, further highlighting its potential as a therapeutic target in high-risk patients [60]. Taken together, these results validate the effectiveness of our risk score model in reflecting the biological pathways that contribute to GBM malignancy.

While the traditional method for stratifying patients by risk score often relies on a median cut-off, we adopted a stricter approach by utilizing quartiles (25% percentile, median, and 75% percentile) to classify patients into low-, medium-, and high-risk groups. This comprehensive perspective allows for more accurate risk stratification and personalized treatment strategies. Given the inherent heterogeneity of GBM and the wide variability in response to current treatments, the use of quartile-based stratification enables a more detailed differentiation between patients, identifying fine risk variations that may be overlooked with a traditional median split. Those in the high-risk group, characterized by significantly poor overall survival and higher resistance to standard therapies like TMZ, can be identified more accurately, facilitating the early consideration of alternative or combined therapeutic strategies.

Several studies have developed gene signatures aimed at improving risk stratification and treatment response prediction in GBM patients. Cao et al. [61] identified four survival-associated DEGs in GBM: *OSMR*, *HOXC10*, *SCARA3*, and *SLC39A10*, with patients in the high-risk group showing poorer survival outcomes. Similarly, Zuo et al. [62] developed a six-gene signature using univariate and multivariate regression models that was an independent prognostic factor, reinforcing its potential clinical application for survival prediction. Wang et al. took a different approach, focusing on angiogenesis-related genes [63]. They identified 31 key angiogenesis-DEGs and established a risk score that proved effective in predicting prognosis and treatment response. Recently, Liang et al. [64] developed a pyroptosis-associated gene signature that stratified patients into high- and low-risk groups, with the high-risk group facing worse survival outcomes. Although these examples have made notable contributions to the field, these previous risk score signatures [61,62,63,64,65,66,67,68] were developed based on older WHO classification systems, which did not account for isocitrate dehydrogenase (IDH) mutation status. The 2021 WHO classification [19] fundamentally changed the landscape of glioma diagnosis by highlighting the importance of IDH mutations in differentiating between various glioma subtypes. IDH-mutant and IDH wild-type gliomas are now understood to have significantly different molecular profiles, biological behaviors, and clinical outcomes [29,69,70,71], making the integration of this marker essential for any current risk assessment tool. By incorporating IDH mutation status classification into our risk score model, we provide a more accurate, up-to-date framework that reflects these advances in glioma classification.

Our model provides valuable insights into alternative therapeutic options for GBM patients, which is crucial for overcoming resistance to TMZ. By predicting responses to a range of therapeutic agents, our model enables a more personalized yet affordable approach to treatment, potentially guiding clinicians in selecting the most appropriate therapies based on the individual risk profile of the patient. The positive correlation between TMZ ln(IC50) values and risk scores in our model confirms that patients with higher risk scores are more resistant to TMZ. While the resistance to TMZ is expected given the use of the TMZ-resistant GBM cell line (TMZ-r), the predictions made for other drugs are particularly important. Our findings suggest that etoposide and paclitaxel may be valuable options for patients in the high-risk group.

The limited efficacy of immune checkpoint inhibitors (ICIs) in GBM highlights the need for improved biomarkers and targeted approaches. Our model aims to address this issue by incorporating factors related to the immunosuppressive microenvironment, which seems to hamper the effectiveness of ICIs [72]. Our model demonstrated strong predictive power for response to immune checkpoint inhibitors, particularly anti-PD−1 therapies, suggesting that this therapy may not benefit patients in the high-risk group. PD−1 expression levels were elevated in TMZ-resistant GBM cells. This finding agrees with previous reports indicating that PD−1 is not confined to lymphocytes, but is also present in GBM cells [17], as seen in other tumor types [73]. This finding raises the possibility that the intrinsic effects of PD−1 inhibition in GBM cells themselves could be relevant, as shown in studies of lung cancer patients [74,75,76]. These intrinsic effects, which may influence tumor cell survival and proliferation, should be considered when designing therapeutic strategies. Our findings suggest that anti-PD−1 therapies are unlikely to succeed as monotherapy for high-risk GBM patients, but they may hold potential in combination with other treatments. Targeting both the immune microenvironment and tumor cell pathways could offer a more effective approach to overcoming resistance and improving outcomes.

In a disease where all patients will eventually die, exploring existing alternative drugs is crucial, as it allows for faster clinical translation of potential treatments. By leveraging the safety profiles of already approved drugs for other diseases, we can expedite their testing in GBM, uncovering new therapeutic options for a disease that currently lacks effective treatments. Repurposing drugs offers a cost-effective pathway to addressing the urgent need for new therapies. Incorporating the risk score into this process can help to identify patients less likely to respond to standard treatments, enabling more personalized approaches and improving treatment outcomes by tailoring therapies to individual tumor characteristics and disease progression.

## 5. Conclusions

Our study presents a risk score model based on DEGs associated with poor TMZ response and high PD−L1/PD−1 expression in GBM. This model effectively stratifies patients into risk groups, correlating with overall survival, immune infiltration, and treatment response patterns. Notably, high-risk patients exhibit features linked to immunosuppression and mesenchymal transition, suggesting limited responsiveness to immune checkpoint inhibitors, but potential sensitivity to alternative chemotherapies. While further validation is necessary, our findings provide a framework for refining treatment strategies in GBM.

## Figures and Tables

**Table 1:** Univariate and multivariate Cox regression analyses of the correlation between 33 shared DEGs and overall survival in TCGA.

	Overall Survival			
	Univariate Analysis		Multivariate Analysis	
	HR (95% CI)	*p*-Value	HR (95% CI)	*p*-Value
*COL6A3*	1.189 (1.061–1.332)	0.003	4.794 (1.796–12.797)	0.002
*CD163*	1.124 (0.979–1.292)	0.097	0.515 (0.295–0.901)	0.020
*ABCC3*	1.108 (0.995–1.234)	0.061	1.381 (1.050–1.815)	0.021
*COL3A1*	1.190 (1.053–1.344)	0.005	0.331 (0.115–0.950)	0.040
*THBS1*	1.341 (1.168–1.540)	<0.001	1.620 (1.014–2.590)	0.044
*GBP5*	1.024 (0.0884–1.187)	0.751	0.745 (0.548–1.011)	0.059
*ITK*	1.162 (1.024–1.317)	0.020	1.225 (0.986–1.522)	0.066
*MARCO*	1.166 (1.006–1.352)	0.042	1.677 (0.936–3.003)	0.082
*UBD*	0.980 (0.861–1.115)	0.756	0.748 (0.526–1.064)	0.106
*MMP7*	1.100 (0.941–1.286)	0.232	1.224 (0.948–1.580)	0.121
*IDO1*	1.070 (0.899–1.275)	0.446	1.171 (0.948–1.446)	0.142
*FCGR2C*	1.289 (1.109–1.499)	<0.001	1.479 (0.872–2.509)	0.146
*PTPN22*	1.176 (1.023–1.353)	0.022	0.717 (0.456–1.127)	0.149
*PLA2G2A*	0.989 (0.855–1.144)	0.883	0.818 (0.603–1.110)	0.196
*COL1A1*	1.252 (1.095–1.431)	<0.001	0.516 (0.187–1.422)	0.200
*LYZ*	1.231 (1.058–1.434)	0.007	1.249 (0.884–1.764)	0.207
*MYO1G*	1.361 (1.170–1.582)	<0.001	1.317 (0.786–2.206)	0.295
*IL21R*	1.233 (1.066–1.426)	0.004	1.277 (0.768–2.121)	0.345
*TREM1*	1.058 (0.948–1.180)	0.314	1.231 (0.794–1.908)	0.353
*FCGR2B*	1.316 (1.129–1.533)	<0.001	1.276 (0.761–2.138)	0.355
*GALNT5*	1.126 (0.992–1.279	0.066	0.893 (0.691–1.155)	0.389
*SAA2*	1.027 (0.899–1.173)	0.699	1.113 (0.821–1.508)	0.491
*IL2RA*	1.082 (0.929–1.259)	0.309	0.834 (0.494–1.406)	0.494
*IBSP*	1.131 (0.972–1.317)	0.111	1.125 (0.795–1.593)	0.505
*F13A1*	1.251 (1.068–1.465)	0.005	0.817 (0.449–1.487)	0.507
*RNASE2*	1.092 (0.957–1.246)	0.188	0.885 (0.537–1.458)	0.632
*FPR2*	1.107 (0.954–1.284)	0.181	0.840 (0.405–1.745)	0.640
*CCR2*	1.181 (1.028–1.356)	0.018	1.091 (0.750–1.589)	0.648
*LTF*	1.003 (0.874–1.153)	0.961	0.965 (0.784–1.187)	0.733
*CCL2*	1.160 (1.018–1.322)	0.026	0.974 (0.655–1.449)	0.896
*CCL8*	1.083 (0.941–1.248)	0.267	1.016 (0.754–1.367)	0.919
*AIM1*	1.144 (1.009–1.296)	0.035	0.979 (0.755–1.269)	0.872
*EMR1*	1.189 (1.022–1.383)	0.025	0.982 (0.646–1.492)	0.931

**Table 2:** Univariate and multivariate Cox regression analyses of the correlation between risk score, clinical features, and prognosis in TCGA.

	Progression-Free Interval				Overall Survival			
	Univariate Analysis		Multivariate Analysis		Univariate Analysis		Multivariate Analysis	
	HR (95% CI)	*p*-Value	HR (95% CI)	*p*-Value	HR (95% CI)	*p*-Value	HR (95% CI)	*p*-Value
Risk Score	2.071	0.004	1.877	0.017	2.531	<0.001	2.141	0.003
	(1.256–3.416)		(1.121–3.142)		(1.569–4.082)		(1.298–3.530)	
Gender	1.313	0.0977314	1.216	0.2467	1.128	0.48344	1.017	0.9238035
	(0.951–1.813)		(0.873–1.694)		(0.805–1.580)		(0.716–1.445)	
Age	1.016	0.0146262	10.123	0.0805	1.030	<0.001	1.025	0.002
	(1.003–1.029)		(0.999–1.026)		(1.015–1.046)		(1.009–1.041)	
Karnofsky performance score	0.995	0.4448754	0.998	0.7798	0.982	0.009	0.990	0.1893078
	(0.982–1.008)		(0.984–1.012)		(0.969–0.996)		(0.976–1.005)	

## Data Availability

The data presented in this study were derived from the following resources available in the public domain: Xena Browser: https://xenabrowser.net (accessed on 1 February 2025); ClinicalOmics: https://trials.linkedomics.org/browse/ (accessed on 1 February 2025).

## Supplementary Materials

The following supporting information can be downloaded at https://www.mdpi.com/article/10.3390/biology14050572/s1: Figure S1. Concentration-response curve of chemotherapeutics in U-251 GBM cell line. Human glioblastoma U-251 cells were treated with increasing concentrations of temozolomide, cisplatin, etoposide, and paclitaxel. Cell viability was assessed using the MTT assay, and IC50 values were determined by nonlinear regression analysis using GraphPad Prism software. These IC50 values served as a reference for selecting concentrations used in subsequent single-concentration experiments. Figure S2. Concentration-response curve of temozolomide (TMZ) in U-251 control and TMZ-resistant cell lines. U-251 control cells and TMZ-resistant cells were treated with increasing concentrations of TMZ (0–30 µM) for 72 h, and cell viability was assessed using the MTT assay. The TMZ-resistant cell line exhibited a higher IC50 value compared to the control cell line, indicating increased resistance to TMZ. The IC50 values were calculated using non-linear regression analysis in GraphPad Prism. Figure S3. Flow cytometry analysis of PD−1 and PD−L1 expression in U-251 control and temozolomide-resistant cells. U-251 control and TMZ-resistant cells were stained with an anti-PD−1 FITC antibody. (A) Live cells were gated based on forward scatter (FSC) and side scatter (SSC) to exclude debris and dead cells. (B) Single cells were selected using FSC-A vs. FSC-W to eliminate doublets. (C) PD−1 (FITC) and PD−L1 (APC) expression was analyzed in the gated population. Unstained cells and isotype-matched controls were used to establish gating thresholds and assess non-specific binding. Data was analyzed using FlowJo software. Figure S4. Lack of cytotoxic effect of anti-PD−L1 treatment in temozolomide-resistant GBM cells. U-251 and U-87 TMZ-resistant (TMZ-r) cells were treated with anti-PD−L1 antibody (avelumab, 50 µg/mL), and cell viability was assessed 72 h post-treatment using the MTT assay. Despite increased PD−L1 expression in U-251 TMZ-r cells (Figure S3), treatment with avelumab did not reduce cell viability, indicating that antibody exposure alone does not account for the anti-PD−1–associated viability effects observed in previous experiments. Figure S5: Validation of the risk score model using two independent datasets from the Gene Expression Omnibus (GEO). (A) Risk score distribution in the GSE53373 cohort, showing a clear difference between short overall survival (OS) and long OS patients based on the risk score. (B) Risk score distribution across glioma diagnoses (GBM vs. mIDH gliomas) and Kaplan–Meier survival curves in the GSE43378 cohort. As expected, GBM patients exhibited higher risk scores than mIDH glioma patients. Furthermore, among GBM cases, high-risk patients showed significantly poorer overall survival (*p* < 0.05, log-rank test), supporting the prognostic relevance of the model. Table S1. Classification of TCGA GBM patients based on temozolomide (TMZ) response and expression levels of PD-1 and PD-L1. Table S2. Differentially expressed genes (DEGs) between TCGA GBM samples with lower and higher temozolomide (TMZ) response. Table S3. Differentially expressed genes (DEGs) between PDCD1_high_ and PDCD1_low_ TCGA GBM samples. Table S4. Differentially expressed genes (DEGs) between CD274_high_ and CD274_low_ TCGA GBM samples. Table S5. Gene set enrichment analysis (GSEA) results comparing high- vs. low-risk score TCGA GBM samples.
