# Supplementary figures and images for: Predicting Treatment Outcomes in Glioblastoma: A Risk Score Model for TMZ Resistance and Immune Checkpoint Inhibition
Source: Biology (Basel). 2025 May 20;14(5):572. doi: 10.3390/biology14050572 (PMC12109283; doi:10.3390/biology14050572)

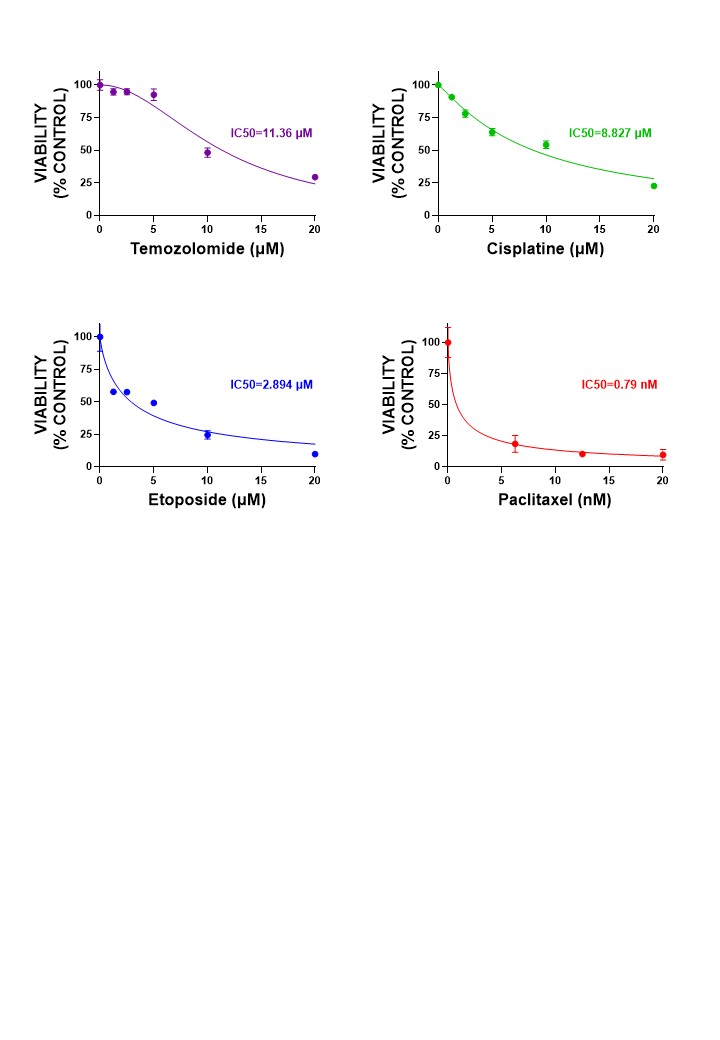

Supplement: Supplementary file 1 [file biology-14-00572-s001.zip › figS1.jpg]

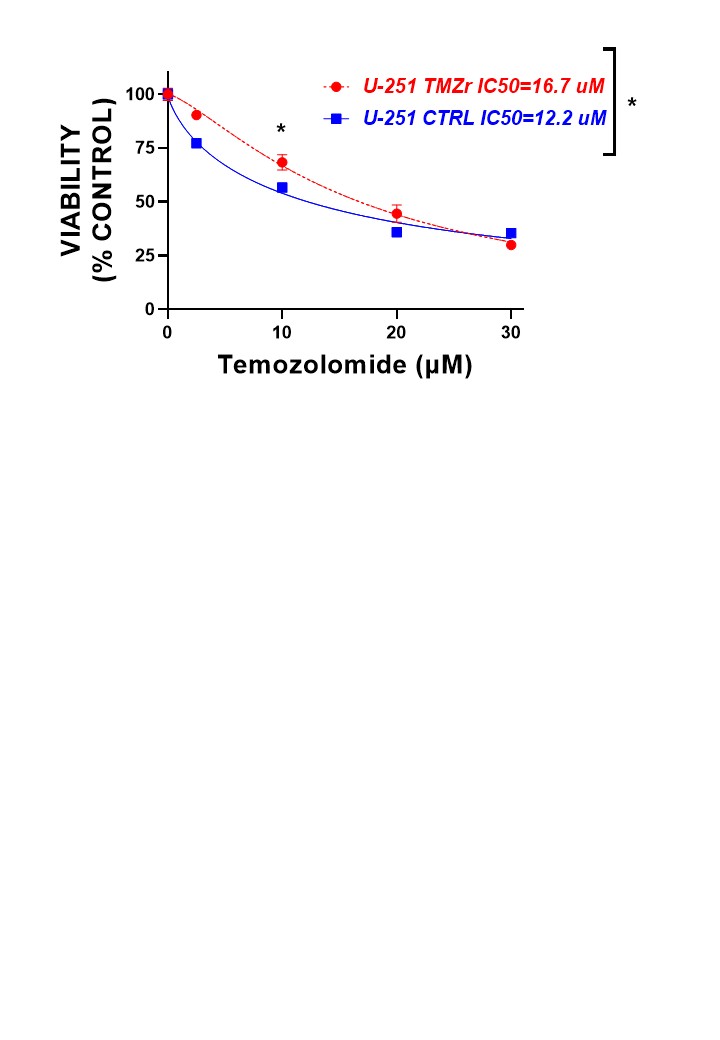

Supplement: Supplementary file 1 [file biology-14-00572-s001.zip › figS2.jpg]

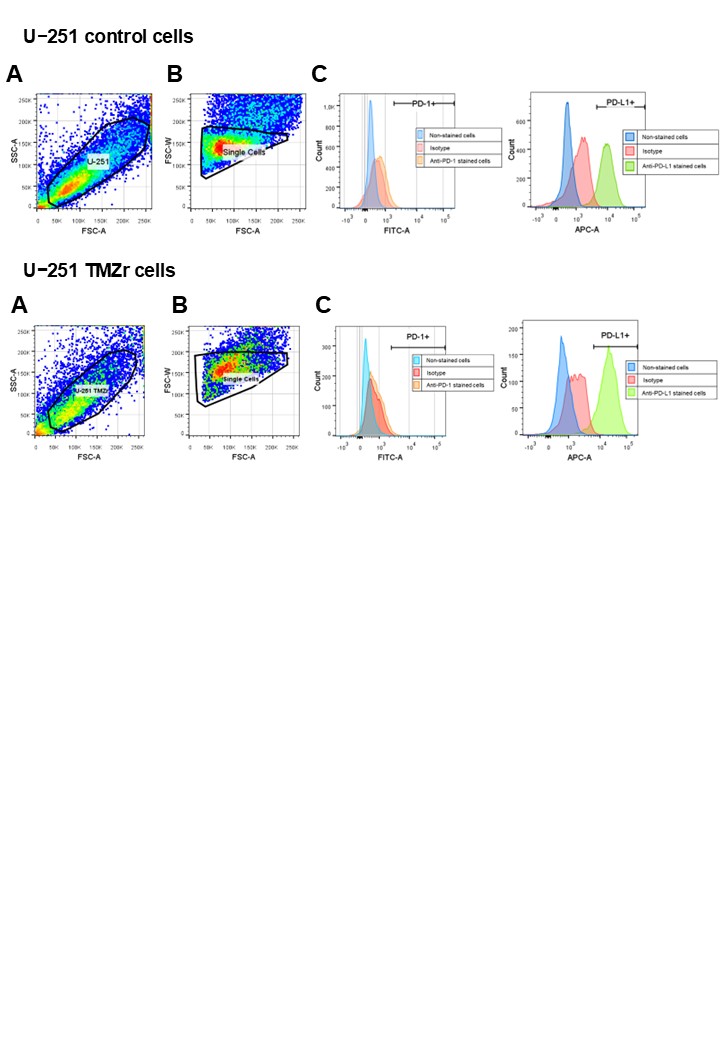

Supplement: Supplementary file 1 [file biology-14-00572-s001.zip › figS3.jpg]

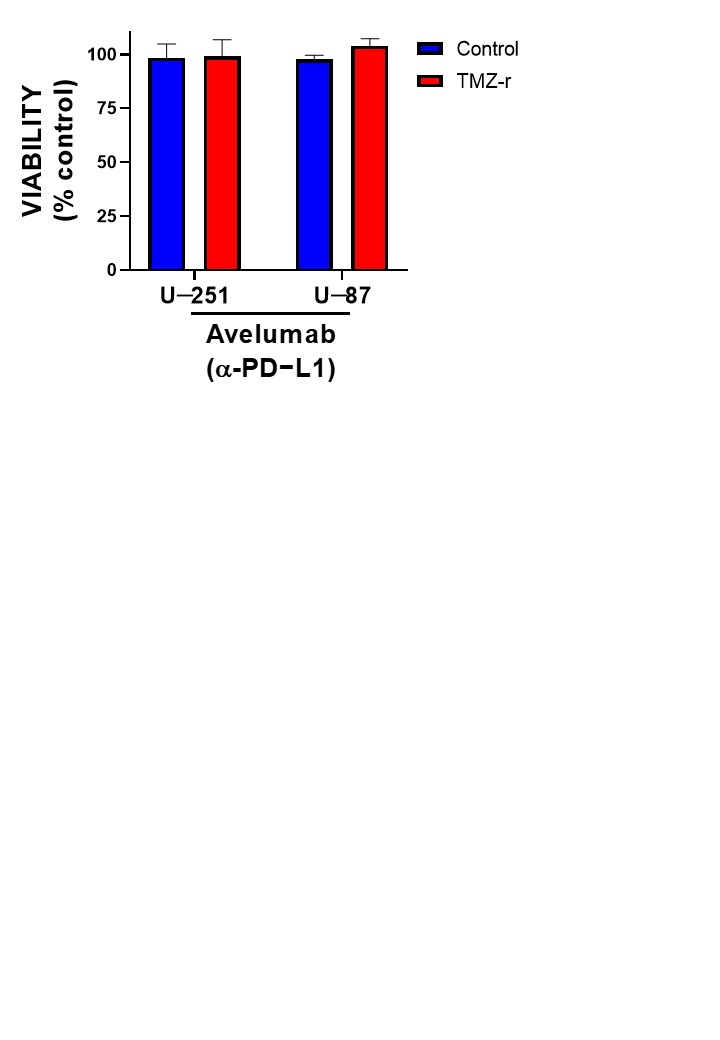

Supplement: Supplementary file 1 [file biology-14-00572-s001.zip › figS4.jpg]

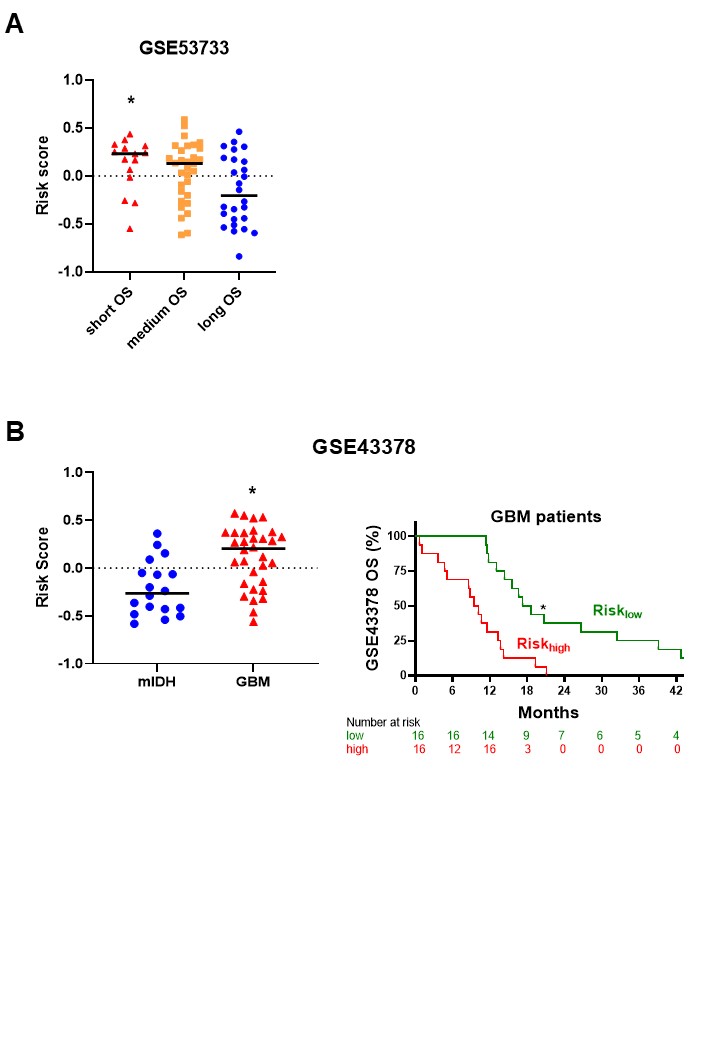

Supplement: Supplementary file 1 [file biology-14-00572-s001.zip › figS5.jpg]
